# Soy, Red Clover, and Isoflavones and Breast Cancer: A Systematic Review

**DOI:** 10.1371/journal.pone.0081968

**Published:** 2013-11-28

**Authors:** Heidi Fritz, Dugald Seely, Gillian Flower, Becky Skidmore, Rochelle Fernandes, Sarah Vadeboncoeur, Deborah Kennedy, Kieran Cooley, Raimond Wong, Stephen Sagar, Elham Sabri, Dean Fergusson

**Affiliations:** 1 Department of Research & Clinical Epidemiology, Canadian College of Naturopathic Medicine, Toronto, Ontario, Canada; 2 Clinical Epidemiology, Ottawa Hospital Research Institute, Ottawa, Ontario, Canada; 3 Ottawa Integrative Cancer Center, Ottawa, Ontario, Canada; 4 Laboratory Medicine and Pathobiology (LMP), University of Toronto, Toronto, Ontario, Canada; 5 Leslie Dan Faculty of Pharmacy, University of Toronto, Toronto, Ontario, Canada; 6 Juravinski Cancer Centre and Department of Medicine, McMaster University, Hamilton, Ontario, Canada; Massachusetts General Hospital, United States of America

## Abstract

**Background:**

Soy and red clover isoflavones are controversial due to purported estrogenic activity and possible effects on breast cancer. We conducted a systematic review of soy and red clover for efficacy in improving menopausal symptoms in women with breast cancer, and for potential impact on risk of breast cancer incidence or recurrence.

**Methods:**

We searched MEDLINE, Embase, the Cochrane Library, and AMED from inception to March 2013 for human interventional or observational data pertaining to the safety and efficacy of soy and red clover isoflavones in patients with or at risk of breast cancer.

**Results:**

Of 4179 records, we included a total of 131 articles: 40 RCTs, 11 uncontrolled trials, and 80 observational studies. Five RCTs reported on the efficacy of soy for hot flashes, showing no significant reductions in hot flashes compared to placebo. There is lack of evidence showing harm from use of soy with respect to risk of breast cancer or recurrence, based on long term observational data. Soy intake consistent with that of a traditional Japanese diet (2-3 servings daily, containing 25-50mg isoflavones) may be protective against breast cancer and recurrence. Human trials show that soy does not increase circulating estradiol or affect estrogen-responsive target tissues. Prospective data of soy use in women taking tamoxifen does not indicate increased risk of recurrence. Evidence on red clover is limited, however existing studies suggest that it may not possess breast cancer-promoting effects.

**Conclusion:**

Soy consumption may be associated with reduced risk of breast cancer incidence, recurrence, and mortality. Soy does not have estrogenic effects in humans. Soy intake consistent with a traditional Japanese diet appears safe for breast cancer survivors. While there is no clear evidence of harm, better evidence confirming safety is required before use of high dose (≥100mg) isoflavones can be recommended for breast cancer patients.

## Introduction

Breast cancer accounts for almost one third of cancers diagnosed among women. In the United States, there were approximately 288 thousand new cases expected for 2011 [1]. Breast cancer is also the second leading cause of cancer death among women, with nearly 40 thousand attributable deaths expected in 2011 in the US [1]. Dietary interventions are emerging as increasingly important strategies for reducing risk of developing breast cancer or recurrence [2,3]. Among breast cancer survivors, for instance, the Women’s Healthy Eating and Living (WHEL) study found that interventions with a diet high in fruits and vegetables, dietary fibre, and low in saturated fat reduced recurrence by 31% among women without hot flashes compared to the control group [4], and that higher vegetable intake, particularly cruciferous vegetables, may have enhanced the effect of tamoxifen, with a 44% reduction in recurrence [5]. If shown effective, these and other dietary strategies represent an important way for women to reduce their cancer risk, or for breast cancer patients to reduce recurrence and safely augment the effects of cancer treatment. Soy has emerged as a specific food that may reduce breast cancer risk [6], and is among the most commonly used complementary medicines utilized by breast cancer patients seeking to reduce risk of recurrence [7,8]. There remains considerable controversy, however, as to its safety, particularly in breast cancer survivors due to purported estrogenic effects [9]. 

Soy, also known as *Glycine max*, contains the class of phytoestrogens known as isoflavones, specifically, genistein, daidzein, glycitein, biochanin A, and formononetin [9]. Isoflavones resemble 17-beta-estradiol in structure, and as such are able to bind the estrogen receptor (ER) in vitro [10], behaving much as a natural selective estrogen receptor modulator (SERM) [9]. For instance, the soy isoflavones have been found to exert partial ER agonist and antagonist activity depending on local estrogen concentrations, with antagonist properties in concentrations similar to premenopausal levels, and agonist properties with postmenopausal levels [9,11-13]. Much of the present debate around soy stems from conflicting in vitro and animal evidence. Some studies show that soy isoflavones can increase tumor cell proliferation [14], while other studies show the opposite [15-18]. The effects of isoflavones in human systems promise to be equally complex [19]. Furthermore, in about 30% of the population, daidzein can be further metabolized in the gut to equol, a metabolite with higher affinity for ER-ß [20-22]. Finally, in addition to ER mediated activity, soy exerts ER-independent effects, including inhibition of vascular endothelial growth factor (VEGF), and proapoptotic effects; genistein in particular inhibits tyrosine kinase and induces the tumor suppressor protein, PTEN [23-25]. 

Like soy, red clover contains the isoflavones genistein, daidzein, biochanin A, and formononetin, however, soy contains higher amounts of genistein and daidzein, while the dominant isoflavones in red clover are biochanin A and formononetin [26-29]. In vivo, formononetin is metabolized to daidzein [30], which may be metabolized to equol among equol producers [31-33]. There are several commercial extracts of red clover, marketed for the treatment of menopausal symptoms including hot flashes (Promensil®), as well as for menopause related health concerns such as bone loss and dyslipidemia (Rimostil®); Trinovin® is marketed for men’s health, specifically for benign prostatic hypertrophy. Promensil® contains 26mg biochanin A, 16mg formononetin, 1mg genistein, and 0.5mg daidzein per tablet (~40mg total) [27]. Other commercial products such as Rimostil® and Trinovin® contain slightly varying amounts, but are still largely comprised of biochanin A and formononetin [27]. Red clover also contains coumestrol, a coumestan that has been less well characterized, however, it is present in very low amounts such that its net contribution to red clover’s purported estrogenic effect is questionable [30,34]. 

To better elucidate the effect of soy, red clover, or isoflavones from these plants on breast cancer, we conducted a systematic review of soy and red clover as used by breast cancer patients or those at risk of breast cancer, assessing their impact on the risk of primary breast cancer or risk of recurrence. We also assessed the impact of isoflavones on surrogate endpoints for predicting breast cancer risk, including circulating estradiol and effects on estrogen responsive tissues such as the breast, endometrial, and vaginal tissues. Finally, we assessed the efficacy of isoflavones in treating menopausal symptoms in patients who have undergone breast cancer treatment. 

## Methods

### Search strategy

Electronic search strategies were developed and tested through an iterative process by an experienced medical information specialist in consultation with the review team. Using the OVID platform, we searched Ovid MEDLINE®, Ovid MEDLINE®In-Process & Other Non-Indexed Citations, EmbaseClassic+Embase, and AMED (Allied and Complementary Medicine). We also searched the Cochrane Library on Wiley (including CENTRAL, Cochrane Database of Systematic Reviews, DARE, HTA, and NHS EED). The strategy was peer reviewed prior to execution by an experienced information specialist using the PRESS Checklist [35]. No amendments were suggested. 

Strategies utilized a combination of controlled vocabulary (e.g., Soybeans, Phytoestrogens, Breast Neoplasms) and keywords (soy, plant estrogen, breast cancer). Vocabulary and syntax were adjusted across databases. Searches pertaining to soy and soy isoflavones were performed in May 2011 and updated in March 2013; there were no language or date restrictions on any of the searches. Searches pertaining to red clover and red clover isoflavones were performed in October 2011 and updated in December 2012. Additional references were also sought through hand-searching the bibliographies of relevant items. Specific details regarding the searches appear in Table **S1**.

### Inclusion criteria

For inclusion, evidence had to come from clinical trials or observational studies in humans. Human trials had to: a) assess the safety and/ or efficacy of soy or red clover or isoflavones from these plants in breast cancer patients for the purposes of treatment or secondary prevention, or the reduction of side effects associated with chemo- or radiation- therapy; alternately, human trials had to: b) assess the effect of soy or red clover or isoflavones from these plants on risk of primary breast cancer among women without a history of previous breast cancer. Clinical surrogate studies were included if they examined endpoints directly related to breast cancer risk, pathogenesis, or objective markers assessing healthy bodily function such as hematological function in breast cancer patients. All types of breast cancers (carcinoma in situ, invasive breast cancer) were included. 

Observational studies had to report on risk of primary breast cancer or breast cancer recurrence associated with soy or red clover consumption compared with non-consumption, in a prospective or retrospective design. *In vitro* and *in vivo* studies were excluded due to the high risk for confounding and previous work on natural health products (vitamin A) showing a lack of correlation between preclinical and clinical results [36]. Due to the nature of soy as a commonly consumed food and red clover as a non-dietary item, there were limited observational studies of red clover consumption expected or identified. Therefore these studies focus solely on soy. 

### Record screening and selection

First pass record screening was based on title review with second pass conducted on abstracts and/or full texts where uncertainty existed. Reports published in English only were included for full analysis if they met inclusion criteria. 

### Data extraction

We piloted data extraction forms and conducted extraction independently in duplicate to assess inter-researcher reliability (HF, RF, GF, SV). No major inconsistencies in data extraction were found. Both quality and efficacy data were extracted. Extraction sheets were prepared based on the Consolidated Standards of Reporting Trials (CONSORT) statement for clinical trials and the Newcastle-Ottawa scale (NOS) for observational studies [37-39]. RCTs were assessed for bias using the Cochrane Risk of Bias tool[40].

### Outcomes

Data was collected on breast cancer incidence, recurrence, or death; impact on hot flashes in breast cancer patients; adverse events; and impact on blood or urinary hormone levels: estrone (E1), estradiol (E2), estriol (E3), progesterone (P), leutinizing hormone (LH), follicle stimulating hormone (FSH), and sex hormone binding globulin (SHBG). Data was also collected on the impact of soy on hormonally active tissues, including breast tissue, endometrial tissue, vaginal tissue, and cervical tissue, as well as on menstrual cycle length in premenopausal women.

### Statistics

We were unable to pool study findings due to heterogeneity between studies, however we display individual study results graphically via forest plots. Although we did not quantitatively calculate heterogeneity, an informal assessment indicated qualitative incoherence between studies on important parameters, most importantly the type and dose of intervention or exposure, as well as study populations and endpoints used. 

## Results

**Figure 1:**
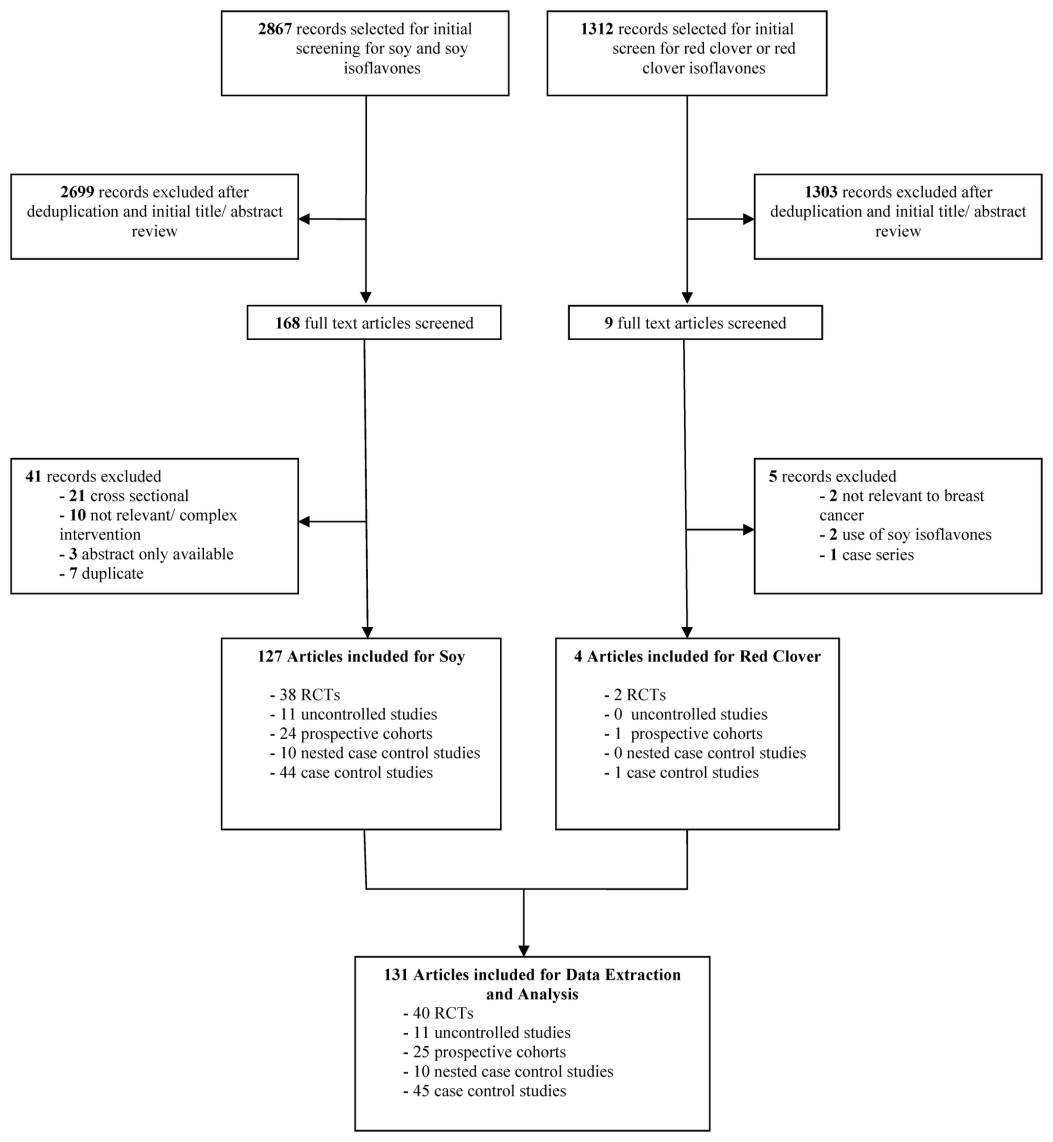
Literature Flowchart.

A total of 4179 records were screened, and 131 records were included: from the soy literature search, 2867 records were screened, of which 127 were included for full analysis and review. From the red clover search, 1312 records were screened, of which four were included for full review. Figure **1** shows a flowchart of the literature search and study selection. We consider the evidence pertaining to soy and red clover independently below.

### Soy

#### Case Control Studies

A total of 44 case control studies pertaining to soy were included. These are described in Table **S2** [41-84]. Of the 44 studies, 30 reported risk ratios and confident intervals relating to the risk of breast cancer; these are displayed in Figures **2**-**3** [42-49,51,52,56,57,61-65,68,70-73,75,76,78,80,81,83,84]. Fourteen studies did not report associations as odds ratios with confidence for overall risk of breast cancer, and so were not included in the figures [41,53-55,58-60,66,67,69,74,79,82,83].

**Figure 2:**
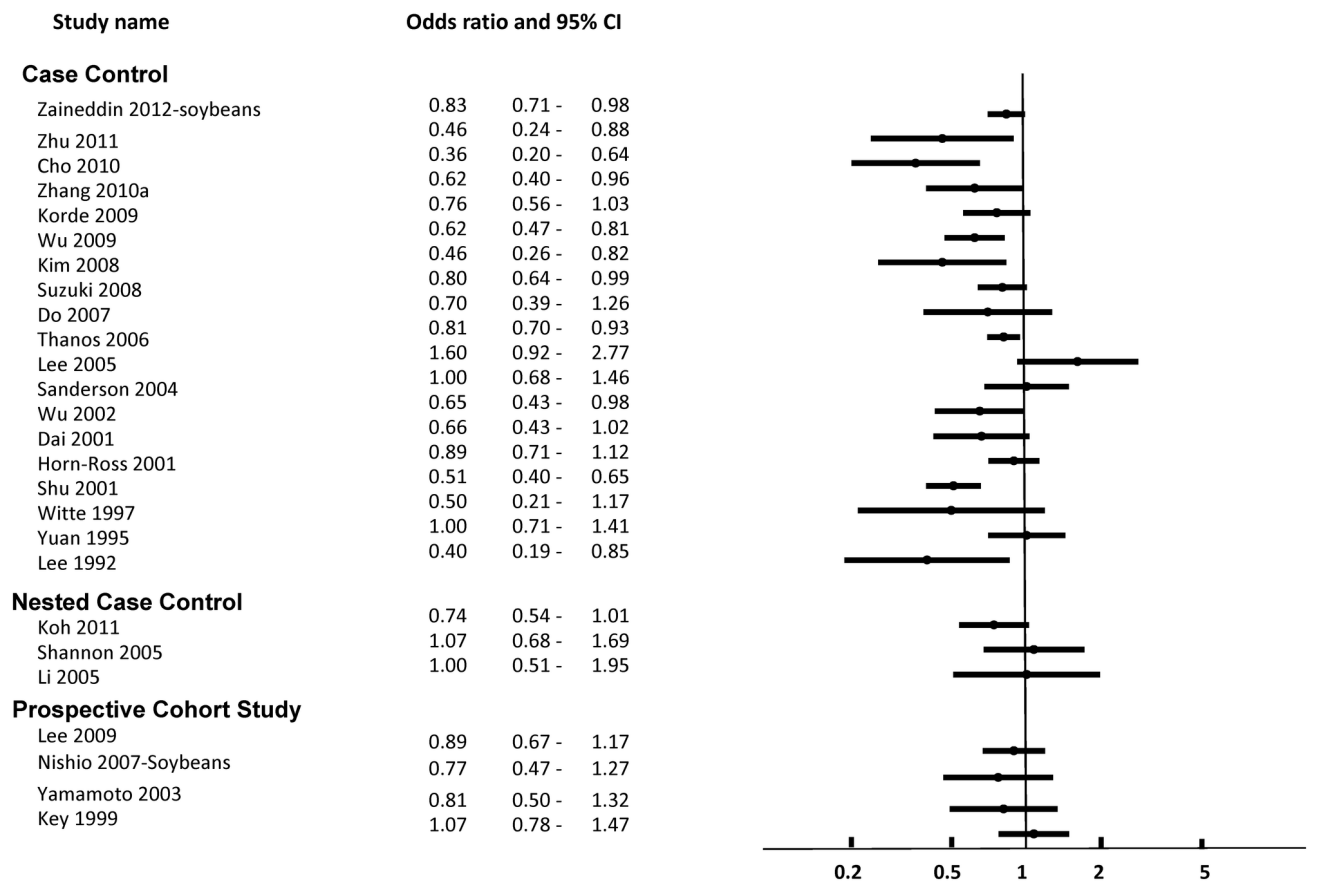
Risk of Breast Cancer Associated with Intake of Soy Food or Soy Protein.

**Figure 3:**
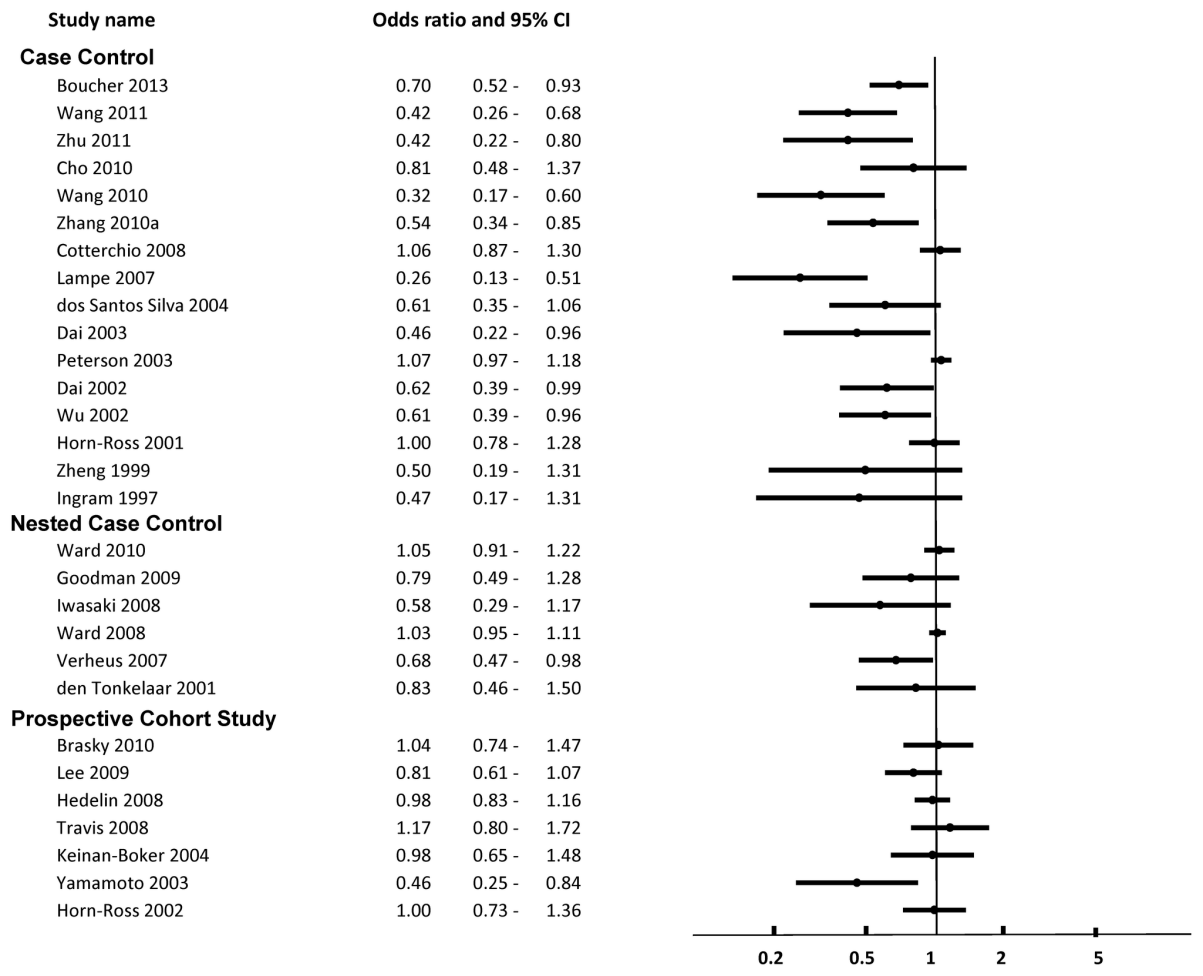
Risk of Breast Cancer Associated with Intake of Soy Isoflavones.

Overall, of the 44 case control studies, 32 showed that higher consumption of soy foods and/ or soy isoflavones was associated with lower risk for primary breast cancer [42-46,49-51,53,54,56-58,61-64,66,71-73,75,77-79,81,83], breast cancer mortality [55], or improved markers of prognosis (ER+ status vs receptor negative status) [74,82] among the overall study population. None of the case control studies examined effects on recurrence or association with use of tamoxifen. None of the studies showed evidence of harm from higher consumption of soy, ie. increased risk of breast cancer or mortality. 

Among studies reporting ORs with confidence intervals, 19 studies described breast cancer risk associated with soy food or soy protein intake [44-46,50,51,56,61,62,64,65,70-73,76-78,80,81], and 16 examined intake of soy isoflavones [42,43,45-49,52,56,57,63,68,75,77,81,84]. Of the 19 studies, 11 (58%) showed significant inverse relationships between soy food or protein intake and risk of breast cancer [44-46,61,64,71-73,77,78,81]. Of the 16 studies, nine (56%) showed a significant inverse relationship between soy isoflavone intake and risk of breast cancer [42,43,45,48,49,63,75,77,81]. There were no studies showing significantly increased risk of breast cancer associated with soy intake.

On subgroup analysis, there was no clear difference in effect based on the type of soy exposure (food / protein, or isoflavones), or according to study quality (NOS score), the measure of exposure (recall or measurement of blood or urinary isoflavone levels), or menopausal status (Figures **S1**-**S2**). There was an indication that higher soy intake, defined as greater than 1 serving of soy food or 6.25g soy protein or 12.5mg isoflavones daily, was associated with a greater protective effect compared to studies examining lower intakes (Figure **S3**). Eight of 12 (67%) studies examining high soy food or protein [45,46,61,64,72,77,78,81] and six of seven (86%) studies examining high soy isoflavones [42,43,45,75,77,81] reported inverse associations between soy and breast cancer risk. On the other hand, two of nine (22%) and zero of three studies respectively found inverse associations with low-medium intake [44,71]. 

Seven studies examined the effect of soy by receptor status; these findings were mixed. Three studies reported no modification of effect by ER/ PR status [46,58,81]. These studies found significant protective effects associated with the use of soy overall, but there was no modification of this effect by receptor status. Anderson et al found that soy isoflavone intake in adulthood was not associated with risk of breast cancer for any receptor type (ER+/PR+, ER-/PR-, or ER+/PR-), but did find that higher intake during adolescence was associated with lower odds of the mixed receptor type: AOR 0.77, 95%CI 0.60-0.99 [41]. Zhang et al found that soy was protective among ER+, ER-, PR+, and PR- tumor types, but the effect was strongest against both ER+/PR+ and ER-/PR- tumors, as opposed to mixed types, ER-/PR+ and ER+/ PR- [83]. Suzuki et al found that soy was significantly protective for ER+, PR+, and Her2- tumors, but not ER-, PR-, or Her2+ tumors [72]. Finally Touillard found that soy was protective for ER+ but not ER- tumors [74]. Five of six studies examining the effects of soyfood consumption in childhood found that higher soy intake, ranging from ≥1 to ≥4 servings per week, compared to lower intake, was significantly protective against breast cancer in adulthood [62,71,73,77,78]. In addition, there was some evidence that soy may be more protective among women with higher waist-to-hip ratio and/ or body mass index, or higher serum estradiol (>5.73 pg/mL) [49]. 

#### Nested Case Control Studies

A total of 10 nested case control studies were included pertaining to soy, shown in Figures **2**-**3**. The nested case control studies were embedded within larger cohorts, which are also reported in their entirety as prospective cohort studies below. These were the Shanghai Breast Self-Exam study, various arms of the EPIC study, and the Multiethnic cohort in Hawaii [85-92]. As with case control studies, these studies primarily assessed the risk of developing breast cancer associated with soy intake, with mixed results. 

Of eight studies assessing risk of breast cancer, one showed significantly reduced risk associated with higher plasma genistein [90]. Grace et al reported a small but significant increase in risk of breast cancer associated with higher serum daidzein and serum and urinary equol levels; this was expressed as a log_2_ odds ratio (associated with doubling of exposure) and as a different measure was not included in the forest plots [88]. Nonetheless, these findings indicated a 30-45% increased odds of breast cancer with a doubling of daidzein (equol precursor) and equol levels. In this study, 39% of the population were equol producers. No significant associations were found for other isoflavones including genistein, glycitein, or O-MDA (O-desmethylangolensin, another daidzein metabolite).

Five studies showed no significant effects in either direction. One study examined mammographic density as a surrogate of breast cancer risk, however with mixed findings [85]. While cases were shown to have higher breast density at all ages compared with control subjects, and this was significantly associated with soy intake during adulthood (-8.6%, p=0.04), the validity of equating this association with increased risk of breast cancer was undermined by associations between body mass index and breast density. Leaner women with BMI <25 had greater percent density, but lower risk of breast cancer. 

#### Prospective Cohort Studies

A total of 24 prospective cohort studies were included, described in Table **1** and Table **S3** [93-116]. Of 11 studies reporting on risk of primary breast cancer, one (9%) showed that soy isoflavone intake was associated with overall reduced risk of breast cancer [116], while the remaining studies showed no associations [99,104,105,107-109,111,113-115,117]. A total of nine unique studies from this group reported odds ratios with confidence intervals, and are shown in Figures **2**-**3** [99,104,105,107-109,111,114,116]. There were no studies indicating a higher risk of breast cancer associated with soy consumption. As with case control studies, there was no clear difference in effect direction when studies were grouped according to study quality (≤5 or >5 NOS score). 

**Table 1:** Prospective Cohort Studies of Soy and Breast Cancer Recurrence and Survival.

Ref	Cohort Name	CohortN	Cases N	Geographic area	Menopause status	Tamoxifen Use?	Anastrozole Use?	Herceptin Use?	Exposure*	High quartile	Study duration	Years f/u**	Outcome
Kang 2012	Mongolia Medical College	288	125	China	Pre and post	Y: 206	NR	NR	Soy protein & IF	>15.78g protein; >35.30mg IF	2004-2011	5-7y	↑Survival
Woo 2012	Korean cohort	339	25	Korea	Pre and post	Y: n=195	NR	Y: n=28	Soyfoods& soy IF	≥65.7g soyfood; ≥15.2mg IF	2007-2008+	32.6mo	↔Recurrence
Zhang 2012	Mongolia Medical College	616	79 (deaths)	China	Pre and post	40-60%	NR	NR	Soy protein & IF	>13.03g protein; >28.83mg IF	2004-2006+	52.1mo	↑Survival
Caan 2011	WHEL	2736	271	USA	Pre and post	~66%	NR	NR	Soy IF	>16.33mg IF	1991-2006	7.3	↔Survival ↔Recurrence
Kang 2010	Harbin, China	524	185 (recur)	China	Pre and post	100% T or A	100% T or A	NR	Soy IF	>42.3mg IF	2002-2008	5.1	↔Survival ↓Recurrence (postM)
Guha 2009	LACE	1954	282	USA	Pre and post	20-40%	NR	NR	Genistein intake	>13.02mg genistein	2000-2008	6.31	↔Recurrence
Shu 2009	SBCSS	5042	534 (recur)	Shanghai	Pre and post	Y: n=2622	NR	NR	Soy protein & IF	>15.31g protein; >62.68mg IF	2002-2009	3.9	↓Recurrence ↑Survival
Fink 2007	Long Island BrCa Study	1210	113 (deaths)	USA	Pre and post	NR	NR	NR	Soy IF	≥7.48mg IF	1996-2002	~6	↑Survival
Boyapati 2005	Shanghai Breast Cancer study	1459	216 (deaths)	Shanghai	Pre and post	NR	NR	NR	Soyfoods	NR	1996-2002	5.2	↔Survival

No significant effect; A anastrozole; IF isoflavones; LACE study Life After Cancer Epidemiology study; postM post-menopausal women; preM pre-menopausal women; SBCSS Shanghai Breast Cancer Survival Study; T tamoxifen; WHEL Women’s Healthy Eating & Living study

* Exposure is dietary unless specified otherwise (ie. supplements)

** Where (~) is used, the follow up period was not reported in the publication, but an estimate was calculated based the time between the end of the recruitment period and data censure/ end of follow-up.

Two studies reported on associations with mammographic density as a predictor of breast cancer risk [102,110]. One study reported an inverse association between soy intake and breast density [110], while the second reported an inverse association between soy intake and breast density among equol producers only [102].

Two studies reported on menopausal symptoms and quality of life among breast cancer patients [93,95]. There was a positive association between soy intake and hot flashes among premenopausal breast cancer patients in one study suggesting relative estrogen antagonism [93], and an indication that soy supplement use was associated with better physical quality of life in a second study [95].

#### Prospective Studies on Recurrence and Survival

Nine prospective studies reported on risk of breast cancer recurrence or mortality [94,96-98,100,101,103,106,112]. These are described in Table **1**. Five of nine reported on breast cancer recurrence [97,100,103,106,112], and seven of nine reported on mortality [94,96,98,100,101,106]. 

Of five studies reporting on recurrence [97,100,103,106,112], two (40%) showed protective effects [106,112]. Four studies reported risk for the overall study population (Figure **S4**), of which one (25%) found significantly decreased risk of breast cancer recurrence or longer disease-free survival (defined as combined relapse or death related to breast cancer) associated with higher soy intake [112]. The fifth study reported results by pre- and post- menopausal status only; this study found lower risk of recurrence among post-menopausal patients, HR 0.67 (95%CI 0.54-0.85) only [106]. A similar association was found for ER+/ PR+ patients and for those on anastrozole [106]. The amount of soy associated with protective effects in these studies was >15.31g soy protein or >62.68mg soy isoflavones [112]; and >42.3mg soy isoflavones (post-menopausal) [106]. Three of the five studies (60%) found non-significant associations [97,100,103], and there were no studies reporting increased risk of recurrence associated with soy intake.

**Figure 4:**
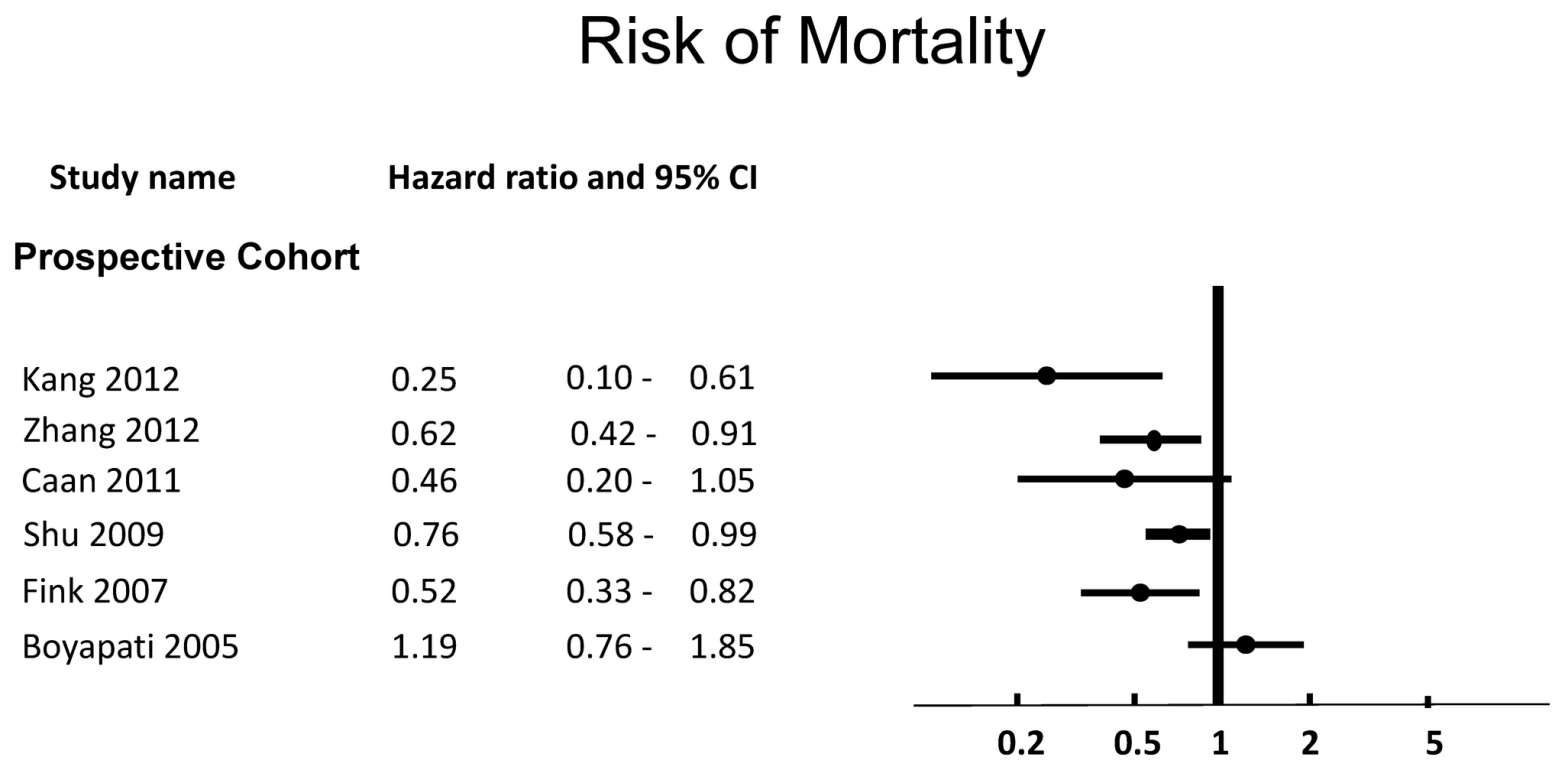
Risk of Mortality Associated with Intake of Soy Protein or Isoflavones.

Of seven studies examining survival [94,96,98,100,101,106,112], four (57%) showed protective effects on breast cancer mortality or mortality in breast cancer patients [94,96,101,112]. Six studies reported overall results for the study population, and are shown in Figure **4**. These effects were seen at a soy intake of >35.3mg soy isoflavones or >15.78g soy protein [94]; >28.83mg soy isoflavones or >13.03g soy protein [96]; >7.48mg soy isoflavones [101]; >62.68mg soy isoflavones or 15.31g soy protein [112]. Caan et al reported a trend toward decreased risk of death among tamoxifen users with intake of total isoflavones ≥6.3mg per day (median 26.7mg), HR 0.26 (0.06-1.01, ptrend=0.05), and also among women with ER+ or PR+ status, HR 0.31 (0.10-0.98, ptrend=0.07), but this was not significant [100]. Two additional studies showed no significant effects [98,106]. 

#### Uncontrolled Trials

**Figure 5:**
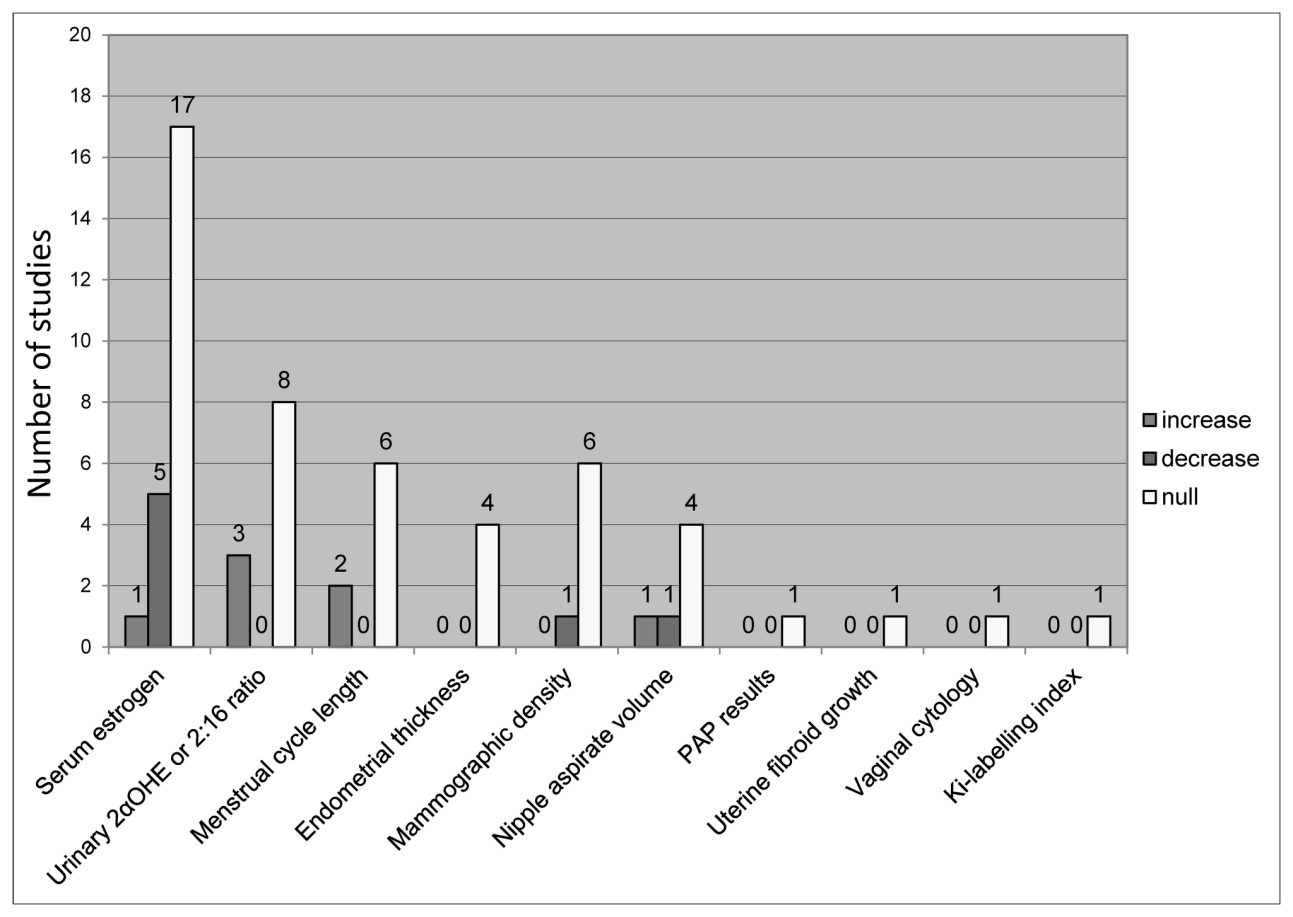
Effect of Soy on Hormonal Biomarkers and Estrogen Dependent Tissues (RCTs and Uncontrolled Trials).

We included 11 uncontrolled trials examining the effect of soy on safety outcomes relevant to breast cancer risk and progression [118,119]. These are summarized in Figure **5** alongside the RCTs, with additional details available in Table **S4**. These studies showed no evidence of harm from consumption of soy. 

In studies conducted among healthy women, there were no significant changes compared to baseline in endometrial thickness [118]; rates of normal mammogram results [118]; menstrual cycle length [120-122]; or urinary excretion of estrogen [123] or the ratio of urinary estrogen metabolites 2:16 OH-estrone (E1) [123,124]. There were varying results with regard to impact on serum estrogen and progesterone levels, with two studies showing no significant impact on serum estradiol [120,125], while two studies show significant decreases in estradiol [121,122], and three studies show decreases in progesterone/ luteal phase progesterone [120-122]. One study indicated that soy may have differential effects in equol producers compared with non-producers: soy consumption increased serum estradiol from 33.75 to 137.4 pmol/L among equol producers, but decreased it from 51.5 to 29.75 in non-producers [119]. With respect to nipple aspirate volume (NAV), a marker of breast tissue proliferative activity, one study showed no change [126] while one study found a significant increase in NAV among premenopausal women only, from 16 to 36 µL [125]. 

#### Randomized Controlled Trials

Of the 38 randomized controlled trials, five assessed the efficacy of soy for hot flashes among breast cancer survivors [127-131], while 34 assessed safety parameters in healthy women [131-164]; one study assessed both hot flashes and safety parameters and hence is counted twice [129]. The five RCTs assessing hot flashes or menopausal symptom scores all found reduced symptoms in both the treatment and the control groups, between 15% to 50% compared to baseline [129,131], however with no significant difference between them. 

A total of 18 RCTs assessed circulating estrogens levels (E1, E2, and/ or E3) Fifteen of 18 studies found no significant impact from soy compared to the control group (p>0.05) [129,132,133,138,140,143,145,147,149,150,155,156,158,160,163]. Three studies found a significant reduction in circulating estrogens compared to the control group (p<0.05) [151,153,155]. One of these found that the consumption of 36oz isoflavone-free soy milk delivering approximately 38g soy protein every day for one menstrual cycle resulted in a 20% decrease in serum estradiol compared to the control group (p<0.03) across the cycle; this was accompanied by a 30% decrease in luteal phase progesterone (p=0.0002), but no change in LH or FSH [151]. One study found that consumption of soy protein (64 or 128g daily for three cycles) resulted in decreases in estrone (E1), testosterone, androstenedione, DHEA, DHEA-s, and cortisol; and increases in progesterone, progesterone: estradiol ratio, FSH and SHBG among equol producers only, compared to non-producers (p<0.05 for all) [153]. Another study by the same group found no significant effect on serum estradiol, FSH, or LH (p>0.05 for all), but a significant decrease in estrone (E1) (p=0.002) and increase in SHBG (p=0.04) compared to control subjects [155]. There were no RCTs showing a significant increase in circulating estrogens. 

A total of nine RCTs assessed the impact of soy consumption on estrogen metabolism as measured through urinary excretion of urinary metabolites. Of these, five reports of six studies found no significant change in 2-hydroxyestrone, 16α-hydroxyestrone, or 2:16α hydroxyestrone ratio compared to the control group (p>0.05 for all) [148,149,156,161,164]. The remaining three studies found significant increases in 2-hydroxyestrone or 2:16α hydroxyestrone ratio [141,152,154], which has been thought to represent an anticancer shift in estrogen metabolism. Nettleton found that consumption of soy protein at a dose of 0.64mg isoflavones/kg daily had no impact on urinary estrogen metabolites in the group as a whole; however there was a significant increase in 2-hydroxyestrone (p=0.01) and 2:16α hydroxyestrone ratio (p=0.04) among subjects who were equol producers [141]. Xu reported a significant decrease in 4-hydroxyestrone (p<0.05) and an increase in 2:16α hydroxyestrone ratio (p<0.05) among subjects consuming soy protein isolate (65 or 132g daily) compared to those consuming a low-isoflavone protein isolate (control) [152]. Lu reported similar results from consumption of 36oz soymilk delivering 158mg isoflavones daily, with significant increases in 2-hydroxyestrone and 2:16α hydroxyestrone ratio compared to subjects consuming an isoflavone-free soymilk [154].

None of the studies assessing endometrial thickness [132,136,155], mammographic density or mammographic changes [66,132,136,137,144,146], nipple aspirate volume [134], PAP results and uterine fibroid growth [132], or vaginal cytology [155] found any evidence of negative impact from soy compared to the control group. Kataoka et al. reported no impact of soy on mammographic density when utilizing two of the three assessment methods; one method found a significant decrease in breast density among the soy group compared to the controls (p=0.04), but this became non significant after adjusting for baseline density [137]. Two of five studies assessing menstrual cycle length reported significant increases of between 1.8 to 3.5 days in cycle length associated with soy consumption compared to control subjects [149,150]; the remaining studies found no effects [147,156,158]. 

Three studies looked at molecular markers of breast cell proliferation or genetic markers of breast cancer risk. One study found no change in BRCA 1 and 2 mRNA level due to soy consumption, however there was a decrease over time in the placebo group, yielding a significant difference between groups (p<0.001) [136]. One study of women with either benign or malignant breast disease, found that consumption of 60g soy protein delivering 45mg isoflavones daily increased in vivo markers of breast epithelial proliferation: thymidine labeling index showing the number of cells in S-phase (p=0.026) and progesterone receptor expression (p=0.04), both increased after two weeks compared to baseline [157]. On the other hand, a recent study found that consumption of an isoflavone supplement (235mg daily) for six months had no effect on Ki-67 labeling index compared to placebo, although there was a significant decrease in both treatment and placebo groups compared to baseline [160]. There was also no change in the rate of atypical cytology. 

#### Risk of bias

RCTs were assessed as having a moderate risk of bias, with inadequate reporting of random sequence generation and allocation concealment (selection bias) in 18 and 32 of 43 unique RCTs, respectively. The majority of RCTs showed low risk of performance bias, detection bias, attrition bias, and reporting bias; description of blinding of participants, blinding of outcome assessment, complete outcome data and reporting was adequate in over 88% of the studies. The risk of bias across studies is shown in Figure **S5**.

#### Adverse Events

The most common adverse event associated with soy consumption was mild to moderate gastrointestinal discomfort, but this usually occurred with comparable frequency in both the soy and the placebo groups [128,130,131,136,142,147,150]; only in one study was there considerable difference in the frequency of GI upset, 47% in the soy group compared to 22% in the control group [130]. Amongst all the studies, there was report of only one case of breast cancer recurrence that occurred in a soy-allocated subject [142]; and two new cases of breast cancer and one case of endometrial cancer among soy allocated subjects compared to five in the control subjects [132,145,147,160]. There were no other serious adverse events. 

#### Interactions

Seven studies included in our review investigated the effect of soy in combination with hormonal therapies: tamoxifen and aromatase inhibitors. Four cohort studies [100,103,106,112] and three RCTs [128,130,131] reported no significant differences with respect to treatment outcomes or rates of adverse effects associated with use of soy among women who were receiving tamoxifen therapy. In one RCT, vaginal spotting was reported in four subjects in the soy group compared to one in the placebo group, but with such low numbers, the statistical significance of this finding was not reported [130]. 

Of the cohort studies, the WHEL cohort exhibited relatively low soy isoflavone intake (≥6.3mg total isoflavones) and yielded null results: HR for recurrence among tamoxifen users was 0.59 (0.27-1.29) [100]. Guha et al found no significant effects overall associated with intake of either ~13mg genistein, ~9.5mg daidzein, or ~800mg glycitein, however there was a trend toward a protective effect among tamoxifen users, p trend =0.05 [103]. Shu et al examined 5042 breast cancer survivors, roughly half of whom were on tamoxifen (n=2262), and found that both soy protein intake (>15.31mg/d) and total isoflavone intake (>62.68mg/d) were protective against recurrence and death, HR recurrence 0.66 (0.52-0.84) for protein, and HR 0.74 (0.59-0.95) for isoflavones [112]. When analyzed by receptor status, these associations remained significant only among women with ER+ status [112]. Kang et al found decreased risk of recurrence among post-menopausal women consuming >42.3mg isoflavones in a cohort of breast cancer patients, all of whom were on tamoxifen or aromatase inhibitors, AHR 0.67 (0.54-0.85) [106]. 

One cohort study examined soy consumption by post-menopausal women who were on anastrozole, and found decreased risk of recurrence among those with an intake of >42mg/d isoflavones, AHR 0.65 (0.47-0.85) [106].

### Red Clover

#### Human trials

Two RCTs pertaining to red clover were included [165,166]. These investigated the effects of the proprietary red clover extract, Promensil®, on hot flashes or estrogen-responsive tissues as a surrogate of breast cancer risk, among high risk populations. 

In brief, Atkinson et al assessed the effect of red clover on mammographic density as well as a panel of other markers of estrogenic activity, in 205 women with an increased risk of breast cancer due to their breast density pattern (Wolfe P2 or DY mammographic breast patterns); there was also a secondary assessment of hot flashes [165]. Participants were randomized to receive 40 mg red clover isoflavones (Promensil®) or placebo daily for one year. 

Powles et al investigated the effect of red clover in 401 women with a family history of breast cancer (at least one first degree relative affected), assessing circulating FSH, endometrial thickness, mammographic density, and bone density [166]. Participants were randomized to receive 40mg red clover isoflavones (Promensil®) or placebo for three years. 

#### Hot flashes

Results of the study by Atkinson showed no significant changes in hot flash score (p=0.88) or mean number of daily hot flashes (p=0.41) when groups were compared [165]. Nonetheless, it should be noted that this was a secondary endpoint, and that this study included women in whom the severity of menopausal symptoms was already low at baseline; menopausal symptoms were not present in all subjects. Baseline mean number of hot flashes per day was 2.1 +/-2.7 for the treatment group and 2.5 +/-3.0 in the control group. There was a comparable decrease of -0.8 +/-2.1 at 12 months for the red clover group and -1.0 +/-1.8 for the control group (p=0.41), which however was not significant. Similar non-statistically significant results were found for the menopausal symptom score, a composite of 21 symptoms including night sweats, palpitations, tension/ nervousness, irritability, insomnia, and mood alterations scored for severity. Adverse events (AE) were not reported.

#### Risk of breast cancer

Neither of the RCTs reported on breast cancer incidence rates, however surrogate markers of estrogenic activity were examined. Atkinson reported no significant changes in estradiol, FSH, or LH over the one year period: estradiol increased by 14.0 pmol/L in the red clover group compared to a decrease of 0.9 in the placebo group, however this was not significant, p=0.49); FSH decreased 4.2 IU/L in the red clover group compared to a decrease of 2.9 in the placebo group, p=0.83; LH decreased 4.0 IU/L in the red clover group compared to a decrease of 4.2 IU/L in the placebo group, p=0.71; and tyrosine kinase increased 1.62 units of activity/ µg protein in the red clover group compared to an increase of 0.90 in the placebo group, p=0.16 [165]. 

Neither of the two RCTs reported any significant changes in mammographic density among both pre- and post-menopausal women [165,166], and Powles found no significant changes in endometrial thickness between groups [166], though neither of these markers are considered to be highly specific or sensitive predictors of breast cancer risk. Atkinson reported a significant interaction between treatment group allocation and the ESR1 polymorphism with respect to effect on mammographic breast density, with decreases of -3.4 (+/-9.7) and -5.2 (+/- 12.0) percent among the CC and CT genotypes respectively, and a 1.4 (+/-12.3%) increase in the TT group (*P* = 0.009) [165]. ESR1 codes for the estrogen receptor α, and variations may be associated with risk of breast cancer [167]. For instance, CC and CT genotypes may be associated with a small reduction in risk of breast cancer compared to TT: CC vs. TT: OR 0.92, 95%CI 0.86-0.99, and CC/CT vs. TT: OR 0.95, 95%CI 0.89-1.00 [167].. It is difficult to predict if or how a possible increase in breast density might affect breast cancer risk among TT carriers.

#### Adverse Events

Atkinson did not report adverse events. Powles reported adverse effects, which most commonly included breast abnormality, “skin related symptoms” (not described), and other minor adverse events, however these were equally distributed between red clover and placebo groups [166].

#### Risk of Bias

According to the Cochrane risk of bias tool, the trial reported by Powles was assessed as having low risk of bias in all of the categories, except one for which information about detection bias was not provided [166]. The trial by Atkinson was assessed as having low risk of bias in approximately half of the categories but failed to provide adequate information to assess for selection bias, detection bias, and attrition bias [165]. Overall, the RCTs were assessed as having low to moderate risk of bias.

## Discussion

The results of our systematic review suggest that there is a lack of real evidence showing that soy increases risk of breast cancer or breast cancer recurrence. This is an important finding given the generally perceived controversial status of soy in relation to breast cancer [168]. Our review suggests that on the contrary, soy consumption may protect against the development of breast cancer [46,75,109,117], and less so, breast cancer recurrence and mortality [106,112], although this is based on observational data only. Larger, long-term trials are needed to better define these effects. In particular, research is needed to more clearly identify possible subgroups of women that may differentially benefit from soy or not, based on receptor status and/ or use of anti-estrogen therapy. In the meantime, since the overall effect of soy, if any, appears to be protective for both breast cancer incidence and recurrence, moderate soy consumption appears to be safe and possibly beneficial for most women. 

Among studies included in our review, case control studies showed a stronger association between soy and reduced risk of breast cancer. As shown in Figures **2** and **3**, case control studies were much more likely to report significant protective associations between soy intake and risk of breast cancer, while prospective studies were less likely to do so. The reasons for this are unclear. Although not shown here, we conducted subgroup analysis according to the method of exposure assessment to assess for the possibility of recall bias. Our analysis showed no clear separation however, between studies utilizing food frequency questionnaires, structured interviews, or objective assessments of blood or urinary isoflavone concentrations. It is possible that cohort studies were not long enough in duration to prospectively capture the true effect of long term soy exposure. 

The effect of soy on hot flashes in breast cancer patients is not clear. RCTs noted some improvements over time, but not in comparison to placebo [127-131]. This finding may be due to the possibility that soy simply does not possess sufficient estrogenic effects to alleviate hot flashes, or due to confounding by concomittant usage of tamoxifen in three of five of these studies [128,130,131]. There is also the possibility that lack of difference between groups may be due to a large placebo effect, since hot flashes are a subjective outcome, and up to a 40% improvement was reported by the placebo group in one study [130]. Finally, it is possible that soy may in fact possess anti-estrogen activity, as suggested by Dorjgochoo et al, who found that higher soy consumption was associated with increased prevalence of hot flashes among premenopausal breast cancer patients [93]. 

Several factors influence the biological activity of soy isoflavones in the body. First, soy isoflavones show selectivity toward ER-ß over ER-α [168-175]. This is important because ERß appears to be associated with antiproliferative, anticarcinogenic effects, while ERα is thought to promote carcinogenesis, and is the form measured clinically in the treatment of breast cancer patients [176,177]. In the breast, ERß is found in ductal and lobular epithelial cells as well as stromal cells, while ERα is found only in epithelial cells and not stroma of the breast [177]. Moreover, ERα is the receptor used to classify ER positive breast cancer, and the one through which tamoxifen exerts its antiproliferative effects [176,178]. Some have suggested that ERß functions as a possible tumor suppressor gene, pointing out evidence that ERß may control ERα-induced proliferation, and that expression is lost in many breast tumors [177,179,180]. If soy preferentially activates ERß, this may explain its chemopreventive effects. 

Secondly, preclinical evidence has shown that under conditions of high estrogen concentration similar to premenopausal levels, soy isoflavones act as ER antagonists, while under conditions of low estrogen comparable to postmenopausal levels, they are ER agonists [12,181,182]. In a study of soy and MCF-7 breast cancer cell growth as assessed in the presence and absence of estradiol, Imhof et al observed “minor proliferation enhancing effects” [18] that occurred “only at unphysiologically low estrogen levels” [18]. According to Imhof, typical in vivo estrogen levels range from 50–400 pM, with even menopausal estrogen levels exceeding 20 pM, the concentration of genistein achieved through supplementation [18]. According to this line of reasoning, at these relative concentrations, the agonistic effects of estrogen would be expected to outweight those of genistein [18]. It would appear that an important component of interpreting these in vitro and in vivo studies is assessing how well they reflect human biological conditions. 

Placed in this context, the clinical data reviewed by our study, which demonstrates a lack of any clear pro-estrogenic effects from use of soy, is quite noteworthy. We found no effects on circulating estradiol, and no measurable effects on estrogen-sensitive target tissues, such as breast tissue (density) and endometrium. In addition, our findings are in agreement with those reported by Hooper et al in a 2009 meta analysis of 47 trials [31]. Hooper found that soy did not significantly affect estradiol, estrone, or sex hormone binding globulin (SHBG), although amongst pre-menopausal women there was a significant 20% decrease in LH and FSH (p=0.01, 0.5 respectively) [31]. In post-menopausal women there were no significant effects on any hormone including estradiol and estrone, with a small nonsignificant 14% increase in estradiol based on 21 studies (p=0.07) and a nonsignificant decrease in estrone. A second meta analysis similarly found no significant effects from soy on breast density, also considered a surrogate of breast cancer risk [183]. 

Despite this, and despite the fact that our study failed to show any clear estrogenic effects observable in humans overall, the possibility of soy having estrogen-like effects under some circumstances in certain subgroups of women cannot be ruled out [184]. Of particular concern is the use of soy among women who are receiving antiestrogen therapy. Tamoxifen acts primarily through the ERα while soy is preferential toward ERß, which theoretically suggests minimal risk of interaction based on competitive receptor binding, however this has not been tested clinically [176,178]. Some preliminary evidence indicates that isoflavones may have synergistic effects with tamoxifen in cancer models and may reduce the development of tamoxifen resistance [185,186]. The in vitro receptor binding activity of genistein, daidzein, equol, and their metabolites is approximately 3% or less that of estradiol for ERα, and 18% or less for ERß [187], while the relative transactivation activity of these isoflavones ranged from 55 to 84% relative to estradiol, compared to 43 to 55% for tamoxifen for both ERα and ERß [187]. Nonetheless, caution should be used with using soy alongside tamoxifen until there is clinical data demonstrating the safety. 

Our review revealed no clear modification of soy’s effect based on menopausal status or ER+/- status in breast cancer patients in large observational studies. However we did find variation according to geographical locale; studies conducted in Asian countries more often reported chemopreventive effects compared to studies in Western countries, which more often reported null results. We attribute this to a difference in soy consumption between these areas. The traditional Japanese diet contains between 6-11g of soy protein and 25-50mg isoflavones; top percentiles of soy intake in Asian studies consume as much as 20g soy protein or 100mg isoflavones per day [188], while the cut off for the top quartiles of intake among Western populations is in the range of a few hundred micrograms (mcg) per day [52,74]. 

The suggestion has been made that the effect of soy depends on genetic variations present in Asian populations. Nechuta et al conducted an analysis of the effect of soy on breast cancer recurrence, investigating the possibility of ethnic variations [189]. They reported that in cohorts of American women, after elimination of women of Asian-American descent, the inverse association between soy and breast cancer recurrence remains, undermining the suggestion that the effect of soy is dependent upon genetic difference between ethnicities [189]. 

### Strengths

Our review is broad in its scope, assessing soy in the context of breast cancer from several perspectives, including risk of breast cancer, risk of recurrence, estrogenic effects, and risk of interactions with tamoxifen and other hormonal therapies. Our findings are generally in agreement with those of the American Cancer Society, suggesting that moderate amounts of soy intake (up to 3 servings per day) is likely safe for consumption by women with breast cancer [190]. 

### Limitations

Although we included a large number of studies regarding soy and breast cancer risk, we were unable to pool data with respect to risk of breast cancer and risk of recurrence due to heterogeneity. There is a lack of long term interventional data assessing cancer risk. This is a particularly important shortcoming because RCTs of under 2 years duration are unlikely to reveal any serious adverse effects in breast cancer survivors, including possible interactions between soy and tamoxifen. This question deserves high priority for future research in this area. In addition, there is a need to more carefully assess the dose-response relationship between soy intake and risk of breast cancer in order to more clearly delineate the threshold of exposure needed for potential therapeutic effects. 

### Conclusions

Soy does not appear to influence levels of circulating estrogen or exert estrogen-like effects at target tissues. There is a lack of evidence showing clear effects of soy consumption or supplementation on reduction of hot flashes in breast cancer patients. Observational data suggest that higher soy intake, consistent with that of a traditional Japanese diet, may be protective against the development of breast cancer as well as breast cancer recurrence and mortality, although there is a need for clinical studies to confirm this relationship. Until there is more data supporting safety, caution is warranted with high dose isoflavone supplements in patients with breast cancer. 

## Supporting Information

Figure S1 **Risk of Breast Cancer By Study Quality (Isoflavones).**
(TIF)

Figure S2 **Risk of Breast Cancer by Menopausal Status (Isoflavones).**
(TIF)

Figure S3 **Risk of Breast Cancer by Dose of Soy Isoflavones.**
(TIF)

Figure S4 **Risk of Breast Cancer Recurrence with Intake of Soy Protein or Isoflavones.**
(TIF)

Figure S5 **Risk of Bias Across Studies.**
(TIFF)

Table S1 **Medline Search Strategy.**
(DOCX)

Table S2 **Case Control Studies of Soy and Risk of Breast Cancer.**
(DOC)

Table S3 **Prospective Cohort Studies of Soy and Risk of Primary Breast Cancer.**
(DOC)

Table S4 **Trials Assessing Effect of Soy on Hormonal Biomarkers and Estrogen Dependent Tissues.**
(DOC)

Checklist S1 (DOC)
