# Supplementary figures and images for: Soy, Red Clover, and Isoflavones and Breast Cancer: A Systematic Review
Source: PLoS One. 2013 Nov 28;8(11):e81968. doi: 10.1371/journal.pone.0081968 (PMC3842968; doi:10.1371/journal.pone.0081968)

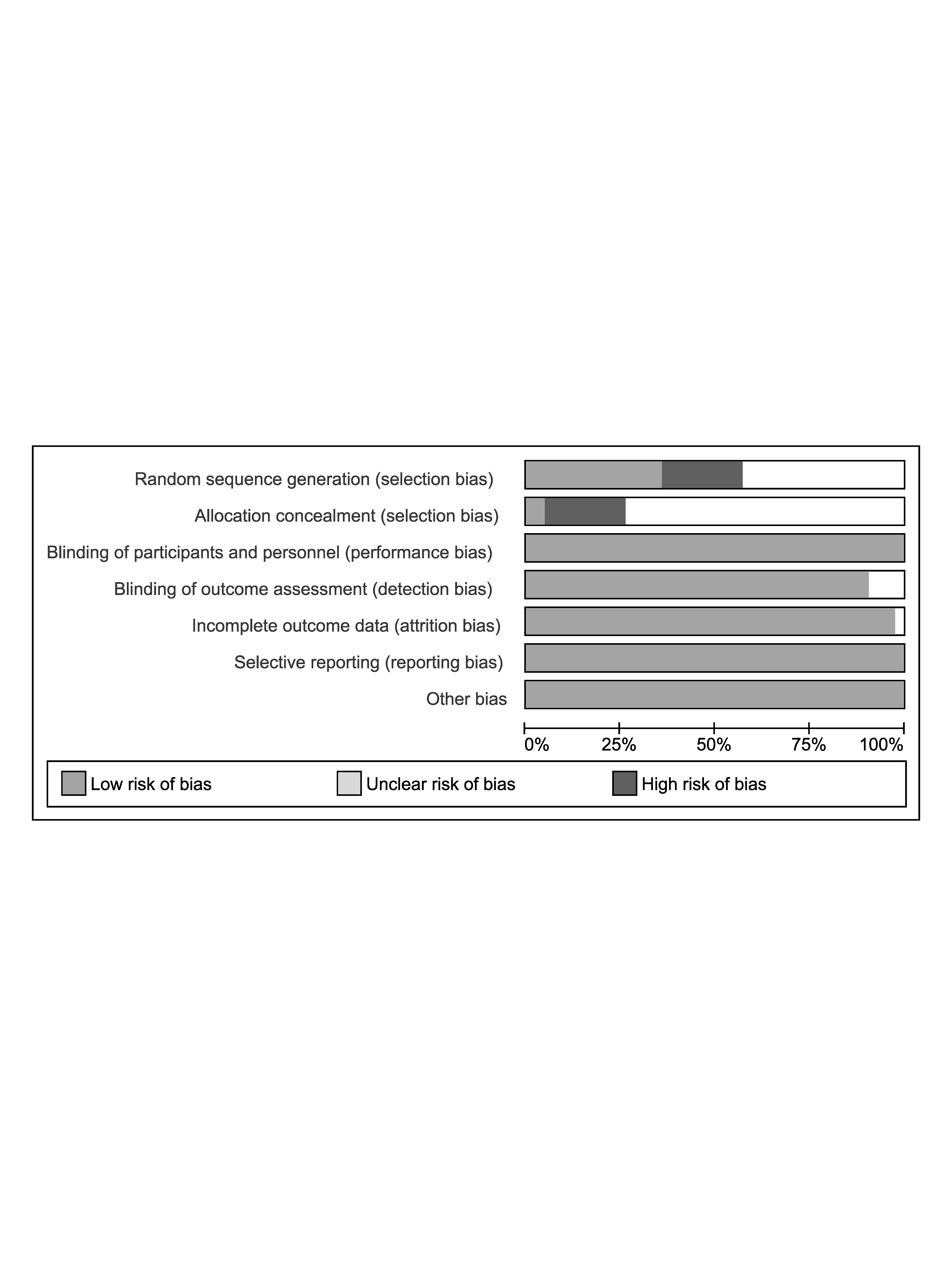

Supplement: Figure S5 — Risk of Bias Across Studies. (TIFF) [file pone.0081968.s005.tiff]
