# Supplementary material for: Soy, Red Clover, and Isoflavones and Breast Cancer: A Systematic Review
Source: PLoS One. 2013 Nov 28;8(11):e81968. doi: 10.1371/journal.pone.0081968 (PMC3842968; doi:10.1371/journal.pone.0081968)
Supplement: Table S1 — Medline Search Strategy. (DOCX) [file pone.0081968.s006.docx]

**Supplemental Table S1. MEDLINE Search Strategy**

| 1 exp Isoflavones/ (16046) |
| --- |
| 2 (isoflavone* or isoflavonoid* or isomil or homoisoflavone* or homo-isoflavone*).tw. (11354) |
| 3 exp Soybeans/ (34869) |
| 4 ("Glycine max" or "glycine max." or "G.max" or "G. max" or "G.max." or "G. max.").tw. (5330) |
| 5 (soy or soybean* or soya or shoyu or soja or sojabohne* or Supro).tw. (65808) |
| 6 (tempeh or tofu or "Ta-tou" or "Ta tou" or Touchi or miso or natto or okara or textured vegetable protein* or texturized vegetable protein* or TVP or yuba or equol).tw. (4271) |
| 7 (bowman-birk inhibitor* or BBI).tw. (931) |
| 8 exp Genistein/ (15615) |
| 9 (genistein or genistin or daidzein or edamame or ononin or formononetin or glycitein or "biochanin A").tw. (18371) |
| 10 exp Phytoestrogens/ (10858) |
| 11 (phytoestrogen* or phyto-estrogen* or (plant adj estrogen*)).tw. (6090) |
| 12 or/1-11 (104556) |
| 13 exp breast neoplasms/ (464924) |
| 14 ((breast$1 or mammar$3) adj2 (neoplasm$ or cancer$ or tumour$ or tumor$ or carcinoma$ or malignan*)).tw. (384174) |
| 15 13 or 14 (531635) |
| 16 12 and 15 (4245) |
| 17 limit 16 to (comment or letter or editorial) [Limit not valid in Embase,AMED; records were retained] (131) |
| 18 16 not 17 (4114) |
| 19 limit 18 to human [Limit not valid in AMED; records were retained] (3497) |
| 20 19 use mesz (1272) |
| 21 exp isoflavone derivative/ (3992) |
| 22 (isoflavone* or isoflavonoid* or isomil or homoisoflavone* or homo-isoflavone*).tw. (11354) |
| 23 exp soybean/ (34869) |
| 24 ("Glycine max" or "glycine max." or "G.max" or "G. max" or "G.max." or "G. max.").tw. (5330) |
| 25 (soy or soybean* or soya or shoyu or soja or sojabohne* or Supro).tw. (65808) |
| 26 (tempeh or tofu or "Ta-tou" or "Ta tou" or Touchi or miso or natto or okara or textured vegetable protein* or texturized vegetable protein* or TVP or yuba or equol).tw. (4271) |
| 27 (bowman-birk inhibitor* or BBI).tw. (931) |
| 28 exp GENISTEIN/ (15615) |
| 29 exp DAIDZEIN/ (3311) |
| 30 exp ONONIN/ (103) |
| 31 exp GLYCITEIN/ (494) |
| 32 exp biochanin A/ (829) |
| 33 (genistein or genistin or daidzein or edamame or ononin or formononetin or glycitein or "biochanin A").tw. (18371) |
| 34 exp phytoestrogen/ (10858) |
| 35 (phytoestrogen* or phyto-estrogen* or (plant adj estrogen*)).tw. (6090) |
| 36 or/21-35 (101307) |
| 37 exp breast tumor/ (464043) |
| 38 ((breast$1 or mammar$3) adj2 (neoplasm$ or cancer$ or tumour$ or tumor$ or carcinoma$ or malignan*)).tw. (384174) |
| 39 37 or 38 (531635) |
| 40 36 and 39 (4230) |
| 41 limit 40 to (editorial or letter) (123) |
| 42 40 not 41 (4107) |
| 43 human.sh. (12483454) |
| 44 (nonhuman or animal or animal experiment).sh. (5625139) |
| 45 44 not (43 and 44) (4558630) |
| 46 42 not 45 (3813) |
| 47 46 use emcz (2302) |
| 48 exp Isoflavones/ (16046) |
| 49 (isoflavone* or isoflavonoid* or isomil or homoisoflavone* or homo-isoflavone*).tw. (11354) |
| 50 exp soybeans/ (34869) |
| 51 ("Glycine max" or "glycine max." or "G.max" or "G. max" or "G.max." or "G. max.").tw. (5330) |
| 52 (soy or soybean* or soya or shoyu or soja or sojabohne* or Supro).tw. (65808) |
| 53 (tempeh or tofu or "Ta-tou" or "Ta tou" or Touchi or miso or natto or okara or textured vegetable protein* or texturized vegetable protein* or TVP or yuba or equol).tw. (4271) |
| 54 (bowman-birk inhibitor* or BBI).tw. (931) |
| 55 (genistein or genistin or daidzein or edamame or ononin or formononetin or glycitein or "biochanin A").tw. (18371) |
| 56 exp phytoestrogens/ (10858) |
| 57 (phytoestrogen* or phyto-estrogen* or (plant adj estrogen*)).tw. (6090) |
| 58 or/48-57 (102356) |
| 59 exp Breast neoplasms/ (464924) |
| 60 ((breast$1 or mammar$3) adj2 (neoplasm$ or cancer$ or tumour$ or tumor$ or carcinoma$ or malignan*)).tw. (384174) |
| 61 59 and 60 (317463) |
| 62 58 and 61 (2205) |
| 63 62 use amed (24) |
| 64 20 or 47 or 63 (3598) |
| 65 remove duplicates from 64 (2474) |
