# Supplementary material for: Soy, Red Clover, and Isoflavones and Breast Cancer: A Systematic Review
Source: PLoS One. 2013 Nov 28;8(11):e81968. doi: 10.1371/journal.pone.0081968 (PMC3842968; doi:10.1371/journal.pone.0081968)
Supplement: Table S2 — Case Control Studies of Soy and Risk of Breast Cancer. (DOC) [file pone.0081968.s007.doc]

**Supplemental Table 2. Case Control Studies of Soy and Breast Cancer Risk (n=44)**

| Ref | N, cases | N, controls | Geographic center of study | Menopause status | Measure of Exposure | Soy measure (unit) | High quartile (daily dose) | Study duration | Outcome* |
| --- | --- | --- | --- | --- | --- | --- | --- | --- | --- |
| Anderson 2013 | 2438 | 3370 | Canada | Pre and post | FFQ: Soy intake – 2y ago | Soy IF | ≥ 497 µg | 2002-2003 | ↔BrCa risk by ER/PR tumor type |
| Boucher 2012 | 3101 | 3471 | Canada | Pre and post | FFQ: IF supps – 2y ago | Soy IF supplements | >40mg | 2002-2003 | ↓BrCa risk |
| Zaineddin 2012 | 2884 | 5509 | Germany | Post M | FFQ: Soy intake | Soy foods | “high” | 2001-2005 | ↓BrCa risk |
| Wang 2011 | 400 | 400 | China | Pre and post | FFQ: Soy intake | Soy IF | ≥ 16.65mg | 2007-2009 | ↓BrCa risk |
| Zhu 2011 | 183 | 192 | China | Pre and post | FFQ: Soy intake | Soy IF | > 28.83mg | 2008-2011 | ↓BrCa risk |
| Cho 2010 | 358 | 360 | Korea | Pre and post | FFQ: Soy intake-previous year | Total soy food | ≥ 122.2 g | 2007-2008 | ↓BrCa risk |
| Wang 2010 | 176 | 176 | China | Pre and post | FFQ: Soy intake – “long term” | Soy IF | ≥ 23.64mg | 2007-2009 | ↓BrCa risk |
| Zhang 2010a | 438 | 438 | China | Pre and post | FFQ: Soy intake- previous year | Soy IF | ≥16.89mg | 2007-2008 | ↓BrCa risk |
| Zhang 2010b | ER+: 431 | ER-: 325 | China | Pre and post | FFQ: Soy intake- previous year | Soy IF (TI, D, G, Gly) | >21.75mg | 2004-2005 | ↑Risk of ER+/PR+ status  (better prognosis) |
| Iwasaki 2010 | 846 | 846 | Brazil, Japan | Pre and post | FFQ: Soy intake - current | Soy IF | NR | 2001-2006 | Interaction b/w soy and SNPs |
| Korde 2009 | 597 | 966 | USA | Pre and post | FFQ: Soy intake - adult, childhood, adolescent intake | Soy intake (svg/wk) | ≥1.11; ≥1.50; ≥2.0 | 1983-1987 | ↔BrCa risk |
| Maskarinec 2009 | 268 | 0 (intra-patient comparisons) | USA | Pre and post | LTSQ: Soy intake – adult, childhood, and adolescent intake | Soy intake (svg/wk) | ≥ 1 | NR | ↔Markers of proliferation and hormonal activity |
| Wu 2009 | 1,248 | 1,148 | USA | Pre and post | FFQ: Soy intake - lifetime intake | Soy intake (svg/wk); IF intake (mg/ 1000kcal) | ≥1 svg; >6.24mg | 1995-2001 | ↓BrCa risk |
| Zhang 2009 | 756 | 1,009 | China | Pre and post | FFQ: Soy intake 1 year pre-diagnosis | Soy IF | >25.40mg | 2005-2005 | ↓BrCa risk by ER/PR tumor type (↓all types) |
| Iwasaki 2009a | 850 | 850 | Brazil, Japan | Pre and post | FFQ: Period NR | Mean IF intake (mg/d) | J: 69.1; JB: 42.8; NJB: 15.0 | 2001-2006 | ↓BrCa risk |
| Iwasaki 2009b | 846 | 846 | Brazil, Japan | Pre and post | FFQ: Period NR | Mean IF intake | NR | 2001-2006 | Interaction b/w soy and SNPs |
| Cotterchio 2008 | 3,063 | 3,430 | Canada | Pre and post | FFQ: Soy foods eaten 2 years ago | IF intake | ≥1237µg | 2002-2003 | ↔BrCa risk |
| Kim 2008 | 362 | 362 | Korea | Pre and post | FFQ: Soy intake for a 12m period over previous 3 years | Soy protein | >10.55g | 2004-2006 | ↓BrCa risk |
| Suzuki 2008 | 678 | 3,390 | Japan | Pre and post | FFQ: Soy intake in year prior to dx/entry | Soybean products | ≥51.2 | 2005-2005 | ↓BrCa risk |
| Do 2007 | 359 | 708 | Korea | Pre and post | FFQ: Soy intake 3 years prior to dx | Total soy foods | >28.81g | 1999-2003 | ↔BrCa risk |
| Lampe 2007 | 196 | 1,,002 | China | Pre and post | Plasma IF (D, G) | Plasma D, plasma G (ng/mL) | D: ≥42.092 G: ≥76.954 | 1995-2000 | ↓BrCa risk |
| Ho 2006 | 161 | 174 | Hong Kong | Post | FFQ (by surviving relative) | Total soy foods | ≥ 4 svg/wk | 1997-1999 | ↓BrCa mortality |
| Piller 2006 | 220 | 237 | Germany | Pre | FFQ: Soy intake 12m before dx | Median plasma genistein (nmol/L) | 1966 | 1992-1995 | ↔BrCa risk (preM) |
| Thanos 2006 | 3,024 | 34,20 | Canada | Pre and post | FFQ: adult and adolescent dietary soy intake | IF intake score | ≥ 22 | 2002-2003 | ↓BrCa risk |
| Touillard 2005 | 88 (ER+) | 36 (ER-) | USA | Pre | FFQ: Soy and IF intake 1 year pre-dx | G and D intake (µg/d) | G: >220 D: >130 | 1998-2000 | ↑risk ER+ status (better prognosis) |
| Lee 2005 | 250 | 219 | Taiwan | Pre and post | FFQ: Soy intake 1 year pre-dx | Total soy foods | >341 g/wk | 1996-1999 | ↔BrCa risk |
| dos Santos Silva 2004 | 240 | 477 | UK | Pre and post | FFQ: IF intake 3 years prior to dx | Soy IF | ≥ 470mg | 1995-1999 | ↔BrCa risk |
| Sanderson 2004 | 1,459 | 1,556 | China | Pre and post | FFQ: Soy intake – previous 5 years | Soy protein | ≥ 12.21g | 1996-1998 | ↔BrCa risk |
| Hirose 2003 | 2,385 | 19,013 | Japan | Pre and post | Questionnaire | Tofu and miso soup (svg) | Tofu: ≥ 5/wk; Miso: ≥ 2/day | 1988-2000 | ↓BrCa risk |
| Dai 2003 | 117 | 117 | China | Pre and post | Urine test | Urinary IF (TI, D, G, Gly) (nmol/mgCr) | TI: 26.41 | 1996-1998 | ↓BrCa risk |
| Peterson 2003 | 820 | 1548 | Greece | Pre and post | FFQ: IF intake 1 year pre-dx | NR | NR | 1989-1991 | ↔BrCa risk |
| Dai 2002 | 250 | 250 | China | Pre and post | FFQ and urinary IF | NR | NR | 1996-1998 | ↓BrCa risk |
| Wu 2002 | 501 | 594 | USA | Pre and post | FFQ: Soy intake over adolescence and adulthood | Tofu intake; Soy IF intake (mg/1000kcal) | ≥ 4 svg/wk >12.68mg | 1995-1998 | ↓BrCa risk |
| Dai 2001 | 1,459 | 1,556 | China | Pre and post | FFQ: Soy intake over previous 5 years | Soy protein | >91.0 g/wk | 1996-1998 | ↔BrCa risk |
| Horn-Ross 2001 | 1,272 | 1,610 | USA | Pre and post | FFQ: Soy intake in 1 yr pre-dx | Tofu | ≥ 1 svg/ mo | 1995-1998 | ↔BrCa risk |
| Shu 2001 | 1,459 | 1,556 | China | Pre and post | Structured interview: soy intake ages 13-15 | Total soy food (tofu, soy milk, other soy) | >11.01g | 1996-1998 | ↓BrCa risk |
| Murkies 2000 | 18 | 20 | Australia | Post | Urine test | Urinary IF (D, G) | NR | 1997-1998 | ↓IF level in BrCa patients |
| Zheng 1999 | 60 | 60 | China | Pre and post | Urine test | Urinary IF (TI, D, G, Gly, E, ODMA) (nmol/mgCr) | ≥18.66 | To 1997 | ↔BrCa risk |
| Ingram 1997 | 144 | 144 | Australia | Pre and post | Urine test | Urinary IF (D, E) (nmol/24hr) | D: ≥1300 E: ≥185 | 1992-1994 | ↔BrCa risk |
| Witte 1997 | 140 | 222 | USA | Pre | FFQ: Tofu intake | Tofu intake | ≥ 1 svg/wk | NR | ↔BrCa risk |
| Wu 1996 | 597 | 966 | USA | Pre and post | FFQ: assessment period not reported | Tofu intake | ≥55 svg/yr | 1983-1987 | ↓BrCa risk |
| Hirose 1995 | 1186 | 23,163 | Japan | Pre and post | Questionnaire: assessment period not reported | Miso and tofu intake | Daily | 1988-1992 | ↓BrCa risk |
| Yuan 1995 | 834 | 834 | China | Pre and post | FFQ: assessment period not reported | Soy protein intake | NR | 1984-1985 | ↔BrCa risk |
| Lee 1992 | 200 | 420 | Singapore | Pre and post | FFQ: Soy intake during year prior to interview | Soy protein intake | ≥3.5g | NR | ↓BrCa risk |

**Key**: A anastrozole; B Biochanin A; Cr creatinine; D daidzein; d day; dx diagnosis; E equol; ER+ estrogen-receptor positive; ER- estrogen receptor negative; F formononetin; FFQ: Food frequency questionnaire; G genestein; g grams; Gly glycitein; IF isoflavone(s); J, JB, NJB Japanese, Japanese Brazilian, and non-Japanese Brazilians; LTSQ Lifetime Soy Questionnaire; Mamm. Mammogram; m month; mg milligrams; ODMA O-desmethylangolensin; PR+ progesterone receptor positive; PR- progesterone receptor negative; svg servings; T tamoxifen; wk week; yr year

*Defined as either analysis for soy food/ protein intake or soy isoflavone intake showing a significant effect. For example if one analysis showed an effect and one analysis showed null findings, an effect was registered here.
