# Supplementary material for: Soy, Red Clover, and Isoflavones and Breast Cancer: A Systematic Review
Source: PLoS One. 2013 Nov 28;8(11):e81968. doi: 10.1371/journal.pone.0081968 (PMC3842968; doi:10.1371/journal.pone.0081968)
Supplement: Table S3 — Prospective Cohort Studies of Soy and Risk of Primary Breast Cancer. (DOC) [file pone.0081968.s008.doc]

**Supplemental Table 3. Prospective Cohort Studies of Soy and Risk of Primary Breast Cancer (n=13)**

| Ref | Cohort Name | CohortN | Cases  N | Geographic area | Menopause status | Breast cancer status at baseline | Tamoxifen Use? | Exposure | Measure | Exposure before cancer? | Study duration | Years f/u* | Outcome |
| --- | --- | --- | --- | --- | --- | --- | --- | --- | --- | --- | --- | --- | --- |
| Brasky 2010 | VITAL | 35 016 | 880 | Washington state | Post | Never | N/A | Soy sup-plements | Questionnaire | before | 2000-2007 | 6 | ↔BrCa risk |
| Lee 2009 | Shanghai Women’s Health study | 73 223 | 592 | Shanghai | Pre and Post | Never | N/A | Soyfoods | FFQ | before | 1996-2005 | 7.4 | ↔BrCa risk |
| Wang 2009 | Women’s Health study | 38 408 | 3234 | USA | Pre and Post | Never | N/A | Tofu | FFQ | before | 1992-2007 | 11.5 | ↔BrCa risk |
| Hedelin 2008 | WLHS | 45 448 | 1014 | Sweden | Pre and Post | Never | N/A | Dietary isoflavone D, G | FFQ | before | 1991-2004 | ~8 | ↔BrCa risk |
| Travis 2008 | EPIC- Oxford | 37 643 | 585: 518 invasive | United Kingdom | Pre and Post | Never | N/A | Dietary isoflavone | FFQ | before | 1993-2003 | 7.4 | ↔BrCa risk |
| Nishio 2007 | JACC | 30 454 | 145 | Japan | Pre and Post | Never | N/A | Soyfoods | FFQ | before | 1988-1997 | 7.6 | ↔BrCa risk |
| Touillaud 2006 | E3N French cohort | 26 868 | 402 | France | Pre | Never | N/A | Dietary isoflavone | FFQ | before | 1993-2002 | ~7 | ↔BrCa risk |
| Nagel 2005 | EPIC- Heidlberg | 54 | 7 | Germany | NR | Never | N/A | Soyfoods, D, G | FFQ | before | 1994-1998 | 3 | ↓Mammographic density |
| Frankenfeld 2004 | Physical Activity for Total Health study | 58 | ** | USA | Post | Never | N/A | Equol producer status | Urinary equol | before | NR | NR | ↓Mammographic density (equol producers) |
| Keinan-Boker 2004 | Dutch arm of EPIC | 15 555 | 280 | Netherlands | Pre and Post | Never | N/A | Dietary isoflavone | FFQ | before | 1993-2001 | 5.2 | ↔BrCa risk |
| Yamamoto 2003 | JPHC study | 21 852 | 179 | Japan | Pre and Post | Never | N/A | Soyfoods | FFQ | before | 1990-1999 | ~9y | ↓BrCa risk |
| Horn-Ross 2002 | California Teachers’ Study | 111 526 | 711 | California | Pre and Post | Never | N/A | Dietary isoflavone | FFQ | before | 1995-1998 | ~3-4 | ↔BrCa risk |
| Key 1999 | Radiation Effects study | 34 759 | 427 | Japan | Pre and Post | Never | N/A | Soyfoods | FFQ | before | 1969-1993 | ~23y | ↔BrCa risk |

**Key**: ↔ No significant effect; A anastrozole; B Biochanin A; D daidzein; E equol; F formononetin; G genestein; Gly glycitein; Mamm. Mammogram; ODMA O-desmethylangolensin; T tamoxifen; TI total isoflavones

* Where (~) is use, the follow up period was not reported in the publication, but an estimate was calculated based the time between the end of the recruitment period and data censure/ end of follow-up.

**This study assessed women for mammographic density according to equol status; there were no breast cancer cases.
