# Supplementary material for: Soy, Red Clover, and Isoflavones and Breast Cancer: A Systematic Review
Source: PLoS One. 2013 Nov 28;8(11):e81968. doi: 10.1371/journal.pone.0081968 (PMC3842968; doi:10.1371/journal.pone.0081968)
Supplement: Table S4 — Trials Assessing Effect of Soy on Hormonal Biomarkers and Estrogen Dependent Tissues. (DOC) [file pone.0081968.s009.doc]

**Supplemental Table 4. Characteristics of RCTs Investigating Soy Surrogates of Estrogenic Effects**

| Ref | N | Random | Control | Blind | Menopause Status | BrCa Status | Geography | Tamoxifen Use? | Intervention Form | Active, Daily Dose | Duration | Effect of Soy on endpoints related to Breast Cancer (c/t control group) |
| --- | --- | --- | --- | --- | --- | --- | --- | --- | --- | --- | --- | --- |
| **RCTs** | | | | | | | | | | | | |
| Khan 2012 | 126 | y | placebo | double | pre/ post | at risk or survivor | USA | n/a | Isoflavone supplement | G 150mg D 74mg Gly 11mg | 6mo | ↔Ki67 labeling |
|  |  |  |  |  |  |  |  |  |  |  |  | ↔Atypical cytology |
|  |  |  |  |  |  |  |  |  |  |  |  | ↔Masood score |
|  |  |  |  |  |  |  |  |  |  |  |  | ↔Hormone levels |
|  |  |  |  |  |  |  |  |  |  |  |  | ↔Gene expression profile |
| Steinberg 2011 OPUS | 403 | y | placebo | double | post | never | USA | n/a | Soy supplement | 80 or 120 mg soy aglycone | 24mo | ↔Hormone levels; |
|  |  |  |  |  |  |  |  |  |  |  |  | ↔Endometrial/ cervical changes; |
|  |  |  |  |  |  |  |  |  |  |  |  | ↔Mammography |
| McLaughlin 2011 | 70 | y x-over | tomato foods | n | post | high risk of BrCa | USA | n/a | Soy protein powder | 50g protein/d | 10wk | ↔Hormone levels |
| *****Maskarinec 2011A,B, 2012, Morimoto 2011, 2012 | 96 | y x-over | low soy diet | n | pre | never | Hawaii | n/a | High soy diet: 2 svg soy/ d | 50mg isoflavones | 6mo | ↓Nipple aspirate volume |
|  |  |  |  |  |  |  |  |  |  |  |  | ↔Hormone levels |
|  |  |  |  |  |  |  |  |  |  |  |  | ↔Urinary estrogen metabolites |
| Maskarinec 2009 OPUS | 358 | y | placebo | double | post | never | USA | n/a | Soy supplement PLUS Ca+D | 80 or 120 mg soy aglycone | 24mo | ↔Mammographic density |
| Marini 2008 | 138 | y | placebo | double | post | NR | Italy | n/a | Soy supplement PLUS Ca+D | Genistein aglycone 54mg | 3y | ↔Mammographic density |
|  |  |  |  |  |  |  |  |  |  |  |  | ↔BRCA 1&2 expression |
|  |  |  |  |  |  |  |  |  |  |  |  | ↔Endometrial changes |
| Kataoka 2008 | 205 | y | placebo | double | NR: 49-65 yoa | never | UK | n/a | Isoflavone supplement 1 tab/d | B 26mg F 16mg G 1mg D 0.5mg | 12mo | ↔ or ↓Mammographic density |
| Pop 2008 | 30 | y | placebo | double | post | never | USA | n/a | Isoflavone supplement 4 cap/d | 600mg “genistein activity” | 84d | ↔DNA/ cellular damage Adverse events |
| Dooley 2006 | 168 | NR x-over | placebo | double | NR | survivors | NR | NR | Isoflavone supplement | 160mg isoflavones | 16wk | ↔Hot flashes |
| DiSilvestro 2005 | 7 | y x-over | placebo | participants blinded | post | survivors | USA | NR | Soy Life Complex supplement 6cap/d | 138mg isoflavones** | 24d | ↔Indirect estrogenic effect (ceruloplasmin activity) |
| Wu 2006 | 57 | y | VLFD or “healthy” control diet | participants blinded | post | never | USA | n/a | 15g soy protein from dietary sources | 50mg isoflavones | 8wk | ↔Hormone levels |
| MacGregor 2005 | 72 | y | placebo | double | NR | pre-existing diagnosis | UK | 78% | Phytosoya® 4 cap/d | 70mg isoflavones | 12wk | ↔Hot flashes |
|  |  |  |  |  |  |  |  |  |  |  |  | ↔Quality of life |
| Nettleton 2005 | 53 | y x-over | milk protein | n | post | survivors + healthy controls | USA | NR | Solae soy protein 0.38g protein/kg /d w/wo probiotics | 0.64mg isoflavones/kg /d | 6wk | ↑Urinary estrogen metabolites (2:16 OH-E ratio) |
| Nikander 2004 | 62 | y x-over | placebo | double | pre/ post | survivors | Finland | n | Isoflavone supplement 6 tab/d | 114mg isoflavones*** | 3mo | Bone markers Adverse events; |
| Zitterman 2004 | 17 | y x-over | soy-free cookies | n | pre | never | Germany | n/a | 5 Soy flour cookies/d | 52mg isoflavones**** | one menstrual cycle | ↔Hormone levels |
| Maskarinec 2004A,B, 2012 | 220 | y | regular diet | n | pre | never | Hawaii | n/a | 2 svg soyfoods per day | 50mg isoflavones | 2y | ↔Mammographic density |
|  |  |  |  |  |  |  |  |  |  |  |  | ↔Hormone levels |
| Nikander 2003 | 62 | y x-over | placebo | double | post | survivor | Finland | n | Isoflavone supplement | 114 mg isoflavonoids | 3mo w 2mo w/o | ↔Hot flashes |
|  |  |  |  |  |  |  |  |  |  |  |  | ↔Hormone levels |
| Maskarinec 2003, 2002A,B | 34 | y | placebo | double | pre | never | Hawaii | n/a | Isoflavone supplement 2 tab per day | 100mg isoflavones^e^ | 1y | ↔Mammographic density |
|  |  |  |  |  |  |  |  |  |  |  |  | ↔Hormone levels |
|  |  |  |  |  |  |  |  |  |  |  |  | ↔Menstrual cycle length |
|  |  |  |  |  |  |  |  |  |  |  |  | ↔Urinary hormones and metabolites |
| van Patten 2002 | 157 | y | placebo | double | post | recently completed tx | Canada | n=38 of 157 | Soy beverage 500 mL/d | 90mg isoflavones | 12wk | ↔Hot flashes |
| Brown 2002 | 26 | y x-over | SAD; ↑PUFA diet | single | pre | never | USA | n/a | Soy diet w 31g soy protein/d | 40mg isoflavones | 2 cycles on, 2w/o | ↔Hormone levels |
|  |  |  |  |  |  |  |  |  |  |  |  | ↔Menstrual cycle length |
| Kumar 2002 | 68 | y | placebo | double | pre | never | USA | n/a | Soy protein powder | 40mg genistein | 12wk | ↔Hormone levels |
|  |  |  |  |  |  |  |  |  |  |  |  | ↑Menstrual cycle length |
| Xu 2000 | 18 | y x-over | low isoflavone protein | NR | post | never | USA | n/a | Soy protein isolate 1.0 or 2.0 mg/kg b.wt | 65 or 132 mg isoflavones | 93d, 26d w/o | ↑Urinary estrogen metabolites (2:16 OH-E ratio) |
| Duncan 2000 | 14 | y x-over | low isoflavone protein | NR | pre | never | USA | n/a | Soy protein isolate 1.0 or 2.0 mg/kg b.wt | 64 or 128 mg isoflavones | 3 cycles, 3wk w/o | ↓Hormone levels (except |
|  |  |  |  |  |  |  |  |  |  |  |  | ↑FSH in equol producers) |
| Lu 2000a | 8 | NR x-over | Isoflavone free soymilk | NR | pre | never | USA | n/a | Soymilk 36 oz/d | ~158 mg isoflavones | 1 cycle, 4mo w/o | ↑Urinary estrogen metabolites (2:16 OH-E ratio) |
| Quella 2000 | 177 | y x-over | placebo | double | pre and post | survivor | USA | 68 women/ grp | Soy supplement, 3 tabs/d | 150mg isoflavones | 4wk, no w/o | ↓Hot flashes |
| Duncan 1999 | 14 | y x-over | placebo –low isoflavone protein | NR | pre | never | USA | n/a | Soy protein powder, 1.0 or 2.0 mg isoflavones /kg b.wt | 65 or 132 mg isoflavones | 93d, 26d w/o | ↓Hormone levels |
|  |  |  |  |  |  |  |  |  |  |  |  | ↔Endometrial proliferation |
|  |  |  |  |  |  |  |  |  |  |  |  | ↔Vaginal cytology |
| Hargreaves 1999 | 84 | y | y | n | pre | y 16 of 84 | UK | n | Soy containing (TVP) bread rolls/d | 45mg isoflavones | 14d pre sx | ↔Breast/ tumor pathology and proliferation markers (except nipple aspirate pS2↑) |
| Martini 1999 | 36 | y | placebo | NR | pre | NR | USA | n/a | Soy protein powder | 38mg isoflavones | 2 cycles | ↔Urinary estrogen metabolites |
|  |  |  |  |  |  |  |  |  |  |  |  | ↔Menstrual cycle length |
| McMichael 1998 | 48 | y | regular diet | n | pre | survivor or at risk | UK | NR | Soy bread rolls, 4/d | 60g protein | 14d | ↑Breast tissue proliferation & activity |
| Nagata 1998 | 60 | y | regular diet | n | pre | never | Japan | n/a | Soymilk, 400mL/d | 109mg isoflavones | 1 cycle | ↓Hormone levels |
|  |  |  |  |  |  |  |  |  |  |  |  | ↔Menstrual cycle length |
| Cassidy 1994 | 6 | NR, x-over | non-soy containing diet | NR | pre | never | USA | n/a | Soy protein supplement 60g/d | 45mg isoflavones | 1mo | ↓Hormone levels |
| **Uncontrolled trials (c/t baseline)** | | | | | | | | | | | | |
| Palacios 2010 | 197 | n | n | n | post | never | International* | n/a | Phytosoya® extract 4 caps/d | 70mg isoflavones | 3y | ↔Mammography; |
|  |  |  |  |  |  |  |  |  |  |  |  | ↔Endometrial changes |
| Hall 2009 | 36 | n | n | n | NR | never | Ireland | n/a | 200mL soy milk, single dose | NR | 1 day | ↓↑Hormone leves (↑equol producers) |
| Maskarinec 2008 | 12 | n | n | n | pre/post | NR | Hawaii | n/a | 2 svg soy milk/d | 50mg isoflavones | 1mo | ↔Nipple aspirate volume |
| Cohen 2007^a^ | 36 | n | n | n | pre | never | USA | n/a | 56.8g soy protein/d | 120mg isoflavones | 4wk | ↔Urinary estrogen metabolites |
| Maskarinec 2005 | 20 | n | n | n | pre | never | Hawaii | n/a | 1 svg soyfood per day | 30mg isoflavones | 8wk | ↔Hormone levels |
| Wu 2000 | 20 | n | n | n | pre | never | USA | n/a | 1 svg soyfoods per day | NR | 3 cycles | ↔Hormone levels |
|  |  |  |  |  |  |  |  |  |  |  |  | ↔Menstrual cycle length |
| Lu 2000b | 10 | n | n | n | pre | never | USA | n/a | Soymilk 36 oz/d | ~154 mg isoflavones | 1 cycle | ↓Hormone levels |
|  |  |  |  |  |  |  |  |  |  |  |  | ↔Menstrual cycle length |
| Petrakis 1996 | 24 | n | n | n | pre and post | NR | USA | n/a | Soy protein isolate | 37.4mg genistein | 6mo | ↔Hormone levels |
|  |  |  |  |  |  |  |  |  |  |  |  | ↑Nipple aspirate volume |
| Lu 1996 | 6 | n | n | n | pre | never | USA | n/a | Soymilk 36oz | ~100mgG +100mgD | 1mo | ↓Hormone levels |
|  |  |  |  |  |  |  |  |  |  |  |  | ↔Menstrual cycle length |

**Legend**: B Biochanin A; b.wt body weight; D daidzein; F formononeitin; G genistein; Gly glycitein; PUFA polyunsaturated fatty acid (diet high in); SAD standard American diet; VLFD very low fat diet; w/wo with or without

* Although from the same cohort, these reports are individually treated in Supplemental table 4 (Results) and highlighted in GRAY.
